# Long-term hyperglycaemia exerts contrasting effects on M1- and M2-like macrophages

**DOI:** 10.3389/fimmu.2025.1639650

**Published:** 2025-07-31

**Authors:** Sona Margaryan, David Poghosyan, Susanna Ghonyan, Lina Hakobyan, Anush Martirosyan, Gayane Manukyan

**Affiliations:** ^1^ Laboratory of Molecular and Cellular Immunology, Institute of Molecular Biology, National Academy of Sciences, Yerevan, Armenia; ^2^ Laboratory of Cell Biology and Virology, Institute of Molecular Biology, National Academy of Sciences, Yerevan, Armenia

**Keywords:** hyperglycemia, GM-CSF and M-CSF-derived cells, M1 and M2 macrophages, phagocytosis, endotoxin tolerance, LPS

## Abstract

**Introduction:**

Chronic hyperglycemia can contribute to metabolic disorders, disrupting cellular homeostasis and potentially leading to immunological disturbances. As highly adaptable innate immune cells, macrophages can effectively utilize glucose for energy and adjust their activities in response to environmental changes. We hypothesized that hyperglycemia induces distinct effects on M1 and M2 macrophages, thereby promoting their divergent roles in the inflammatory response.

**Methods:**

For this, we applied an in vitro hyperglycemia model to investigate its impact on M1- and M2-like macrophages differentiated from primary monocytes.

**Results:**

M1-like macrophages exhibited diminished capacity to produce reactive oxygen species (ROS), IL-6, TNF-α, as well as reduced antigen presentation and co-stimulatory abilities under long exposure to high glucose. In contrast, M2-like macrophages showed a shift toward M1 polarization, characterized by increased production of ROS and IL-6, upregulation of CD86 and HLA-DR expression, and reduced reparative abilities. We also observed disturbance of endotoxin tolerance evidenced by increased production of TNF-α and diminished phagocytic ability.

**Discussion:**

The results suggest that hyperglycemia disrupts the typical functional dichotomy of M1 and M2 macrophages, which may explain mixed polarization of tissue macrophages in individuals with metabolic syndromes associated with chronic hyperglycemia.

## Introduction

Hyperglycemia has been reported to be associated with metabolic diseases and may induce several physiological and pathophysiological changes (1). It is the main reason for the development of complications in both types of diabetics. The progression of tissue damage in diabetes can be attributed, in part, to hyperglycemia-induced disturbance between immune activation and inflammation resolution, where macrophages are capable of participating given their plasticity in response to different stimuli (2). Prolonged hyperglycemic condition results in the development of diabetes mellitus by damaging the pancreatic β-cell and inducing insulin resistance (1). The major cause of comorbidities related to diabetes was proposed to be a release of pro-inflammatory cytokines TNF-α, IL-1β and IL-6 predominantly produced by macrophages (3). Macrophages have an integral role in preserving or damaging pancreatic β-cells with their impact largely determined by their activation state. Given their plastic nature, macrophages can exert multiple roles in the immunity, including activation of the acquired immune response, antigen presentation, wound healing, cytokine/chemokine production thus maintaining immune homeostasis. By simplified classification, macrophages are divided into two major differentiation phenotypes, classically activated macrophages (M1) and alternatively activated macrophages (M2). M1 macrophages secrete IL-6, TNF-α, IL-1β and display functional characteristics that are pro-inflammatory and antimicrobial in nature, whereas anti-inflammatory M2 macrophages secrete IL-10 and other anti-inflammatory factors and performs tissue repair function (4–7).

Hyperglycemia is one of the important factors driving the development of complications linked to diabetes. Notably, even transient episodes of hyperglycemia increase the risk for diabetes-associated complications, termed “hyperglycemic memory”. There is an association between hyperglycemia and the recruitment of monocytes, their infiltration into arterial plaques which contributes to the development of atherosclerosis in animal models (8). Hyperglycemia has already been identified as a direct driver of macrophage dysfunctions (9–12). High glucose levels have been demonstrated to induce reprogramming of macrophages, resulting in reduced inflammatory response, phagocytosis, and nitric oxide production in mouse macrophages (11, 13). In another mouse study, the authors have shown a significantly reduced production of TNF-α and IL-6, low expression of CD86 and CD54, and enhanced nitric oxide secretion by peritoneal exudate macrophages (9).

In the current study, we have applied an *in vitro* model of macrophage differentiation from monocytes in the presence of granulocyte-macrophage colony-stimulating factor (GM-CSF) and macrophage colony-stimulating factor (M-CSF). Macrophages developed in the presence of GM-CSF exhibit a pro-inflammatory M1 phenotype with enhanced antigen presentation and cytokines production, while M-CSF-induced macrophages exhibit immunosuppressive phenotype associated with tissue repair, immune modulation, and production of the anti-inflammatory cytokines IL-10 and a CCL2/MCP-1 (7, 14–16). In this current study, we tested our hypothesis that distinct subsets of macrophages respond differently to hyperglycemia. To test this, we employed an *in vitro* model of human-derived peripheral blood monocytes differentiated into M1- and M2-like macrophage cells. We explored the effects of short- and long-term glucose exposures on the morphology, phenotype and functions, including their reparative and atherogenic abilities, as well as their response to LPS. We also sought to determine how endotoxemia, particularly in the context of hyperglycemia, affects the functionality of M1 and M2 macrophages.

## Methods

### *In vitro* differentiation of primary monocytes in normal and high glucose conditions

Peripheral blood mononuclear cells (PBMCs) were isolated from healthy individuals (age range 25–50 years) without family history of metabolic and cardiovascular diseases. Before enrollment in study, informed consent was obtained from all participants. Ethnic approval was obtained from the ethical committee of Institute of Molecular Biology NAS RA (IRB00004079, IORG0003427). Whitten informed consent was obtained from each participant. Monocytes were purified from the buffy coat using negative selection EasySep™ Direct Human Monocyte Isolation Kit (Stemcell Technologies, USA) according to the manufacturer’s protocol. Isolated monocytes were seeded (0.5x10^6^) in 24-well plate in RPMI-1640 containing normal (10mM) glucose (n-Glu), high (25mM) glucose (h-Glu) or high mannitol (25 mM) as osmotic pressure control, and supplemented with 10% of heat inactivated FBS, 2mM L-glutamine, penicillin (100U/mL), and streptomycin (100 µg/mL) (all from Sigma-Aldrich). To induce macrophage differentiation, monocytes were stimulated with 30ng/mL of GM-CSF or 30ng/mL of M-CSF (BioLegend). On the day of 3 of cell cultivation, the growth medium was replaced with fresh RPMI containing the same glucose concentration and corresponding growth factor. Thereafter, medium replacements were performed every 2^nd^ day. Supernatants from 3, 5 and 7-day cultivations were harvested and stored at -80°C. Macrophages were detached with use of Trypsin and EDTA (Sigma-Aldrich), washed and used for further evaluations.

### Cell viability assay

Apoptotic rate of differentiated macrophages was assessed via Annexin V-FITC (BioLegend) and propidium iodide (PI, BioLegend) double staining by flow cytometry. Annexin V-/PI- cells were considered as viable, annexin V+/PI- cells as early apoptotic, and Annexin V+/PI+ cells as dead cells.

### Oxidative burst assay

To determine oxidative burst capacity of GM-CSF or M-CSF differentiated macrophages, dihydrorhodamine 123 (DHR-123, Sigma-Aldrich) conversion into the fluorophore rhodamine was evaluated. Detached cells were treated with DHR-123 at a final concentration of 10 μM for 1 hour at 37°C. The reaction was stopped by placing the tubes on ice for 10 min. Fluorescence of oxidized DHR123 was measured by flow cytometry.

### Phagocytosis assay

The phagocytic capacity of GM-CSF or M-SCF differentiated macrophages was assessed by using pHrodo™ Green Zymosan BioParticles (ThermoFisher Scientific, MA, USA). Briefly, 25 ug/ml pHrodo BioParticles were added to the macrophage culture for 3 hours at 37°C. Following the incubation, the cells were detached with trypsin-EDTA and washed with PBS. The cells then were placed on ice for 15 min and analyzed by flow cytometry.

### Flow cytometry analysis

To access surface markers expression of GM-CSF or M-CSF-treated monocytes, the cells after detachment were stained with monoclonal antibodies against CD36-FITC (#986302), CD86-PE (#305406), CD206-APC-Cy7 (#321120), CD284-APC (TLR4, #312816) (BioLegend), CD14-APC-Cy7 (#333948), HLA-DR-PE (#568230) (BD Biosciences). For intracellular staining of CD68-PerCP/Cy5.5 (#333813), the cells were fixed with a fixation buffer (BioLegend), washed in PBS and permeabilized using a permeabilization buffer (BioLegend). Isotype controls and/or fluorescence minus one (FMO) controls were employed to ascertain the positivity and negativity of marker expression. The acquisition was done with the LSRII flow cytometer (BD Biosciences) equipped with BD FACSDiva™ v.8.0.1 software. Obtained data were analyzed using FlowJo V10 software (FlowJo, Ashland, OR, USA). The results are presented as the mean fluorescence intensity (MFI) or the percentage of positive events for each marker. Statistical analysis was performed with GraphPad Prism v.5.01 software (GraphPad Software, USA).

### Production of cytokines

Enzyme-linked immunosorbent assay (ELISA) was used to measure the concentrations of IL-1β, IL-6, TNFα, and IL-10 in supernatants of GM-CSF or M-CSF-treated monocytes.

### RT-PCR

To detect mRNA expression of human *NOX2*, *Arginase 1*, and *NF-kB*, total RNA was extracted from cultured cells with TRIzol™ Reagent (ThermoFisher Scientific, MA, USA). One step probe-based real-time RT-PCR kit qPCRBIO Probe 1-Step Go (PCR Biosystems, USA) was used to analyze relative quantities of target genes expression. The expression level of each target gene was normalized to their Actin level using the 2^-ΔCt^ method. Primer sequences used in rRT-PCR experiments were as following: *β-actin* forward 5′-CGCGAGAGAAGATGACCCAGATC-3′ and reverse 5′-GCCAGAGGCGTACAGGGATA-3′; *Arginase-1* forward 5′-ATCAACACTCCCCTGACAACC-3′ and reverse 5′-CGCAAGCCAATGTACACGAT-3′;*NF-κB* p65, forward 5′-CTGTCCTTTCTCATCCCATCTT-3′ and reverse 5′-ACACCTCAATGTCCTCTTTCTG-3′; *NOX2* forward 5′-ACACCCTTCGCATCCATTCTC-3′ and reverse 5′-GCAAACCACTCAAAGGCATGT-3′.

### Fluorescence imaging

GM-CSF or M-CSF-treated monocytes were fixed with Fixation Buffer for 20 min, washed with PBS, then permeabilized for another 20 min. Non-specific binding was blocked with 10% normal donkey serum in PBS for 45 min. This was followed by staining with primary mouse anti-human CD68 antibodies (BioLegend, #333802) overnight at +4°C. Cells were then washed and stained with secondary donkey anti-mouse IgG antibodies conjugated with Alexa Fluor 647 (Abcam, #ab150107) and for cytoskeleton staining of F-actin with Alexa Fluor^®^ 488 Phalloidin (Cell Signaling Technology, #8878) for 45 min. Alternatively, fixed and permeabilized cells were incubated overnight at +4°C with primary rabbit anti-human NOS2 antibodies (MyBiosource, #MBS7122224), followed by staining with donkey anti-rabbit Alexa Fluor 488-conjugated secondary antibodies (Abcam, #ab150073) and Alexa Fluor^®^ 647 Phalloidin (Cell Signaling Technology, #8940). After that cells were washed and stained with Hoechst 33342 (Thermo Scientific) for 5 min and imaged using Cytation C10, Agilent equipped with Gen5 v. 3.14 software.

### Scratch wound healing assay

To evaluate the wound healing capacity of GM-CSF or M-CSF-treated cells, freshly isolated monocytes were differentiated for 5 days as described above. In order to conduct a scratch wound healing model, human epithelial HeLa cells (cervix carcinoma) were grown in a 24-well plate in complete RPMI-1640, until they reached 85-90% confluence. Following this period, the cell monolayer was delicately scratched using a 200μL pipette tip, and supernatants with detached cells were immediately replaced with conditioned media taken from GM-CSF or M-CSF-treated monocytes. Throughout the following incubation period, the progress of wound closure was recorded by imaging reader (Cytation C10, Agilent) at different time points (each 6 hours), and 48h point was chosen as a final time-point. Images were analyzed using Gen5 v.3.14 software. The distance between wound edges was calculated from the multiple measurements. The percentage of wound healing was calculated using a formula:


$$\text{\% }of\;healing=\frac{D0h-D48h}{D0h}*100\%$$

Three independent experiments were performed.

### *In vitro* LDL uptake

Briefly, primary monocytes were differentiated for 5 days as described above and seeded on glass coverslips in 24-well plates. On the 5th day of differentiation, oxLDL (25μg/ml, Invitrogen) was added to the cells for the next 24 hours. Afterward, the cells were washed with PBS, fixed in 4% paraformaldehyde for 30 min and washed again. Cultures were then incubated in 100% propylene glycol for 7 min and stained with 0,5% Oil Red O (Sigma-Aldrich). Unbound dye was removed by rinses with PBS. Cells were mounted with an aqueous mounting medium with DAPI and evaluated by confocal microscopy using Cytation C10 (Agilent) on Cy5 channel. Three independent experiments were performed.

### Induction GM-CSF or M-CSF-treated cells by LPS

Primary monocytes were differentiated into M1- and M2-like macrophages over a period of 5 days, as previously described. After removing the growth factors, 100 ng/ml of LPS was added for the indicated times and the cells were subjected to cytometric and RT-PCR analyses.

### Endotoxin tolerance model

Freshly isolated PBMCs from healthy individuals were cultured in RPMI-1640 and treated with 30 ng/mL GM-CSF and incubated 2 days in 24 well plates, at 37°C with 5% CO_2_. Then medium was replaced and 10ng/mL of LPS was added to corresponding wells and incubated for 20 hours. Following the incubation period, indicated cells were supplemented with 100 ng/mL of LPS and cultivated for an additional 4 hours, to assess LPS tolerance. Detailed sequence of LPS stimulation steps is represented in the experimental scheme. All supernatants were collected and stored at -80°C till cytokine levels measurement. The cells were detached with Trypsin-EDTA solution for further cytometric and functional analyses.

### Statistical analysis

The statistical analysis of obtained data was performed using Graph Pad Prism 9 (GraphPad Software, San Diego, CA). Data is presented as the mean +/- the standard deviation (SD). First, normality of variables was calculated using Kolmogorov-Smirnov D’Agostino test. Data comparison was performed by using one-way repeated measures ANOVA with Tukey’s or Brown-Forsithe multiple comparison *post hoc* test. Analysis between independent data was done with unpaired *t* test or Mann-Whitney test, based on data distribution. A *p*-value <0.05 was considered significant.

## Results

### GM-CSF- and M-CSF-treated monocytes exhibit characteristics reminiscent of M1 and M2 macrophage polarization, respectively

**Figure 1:**
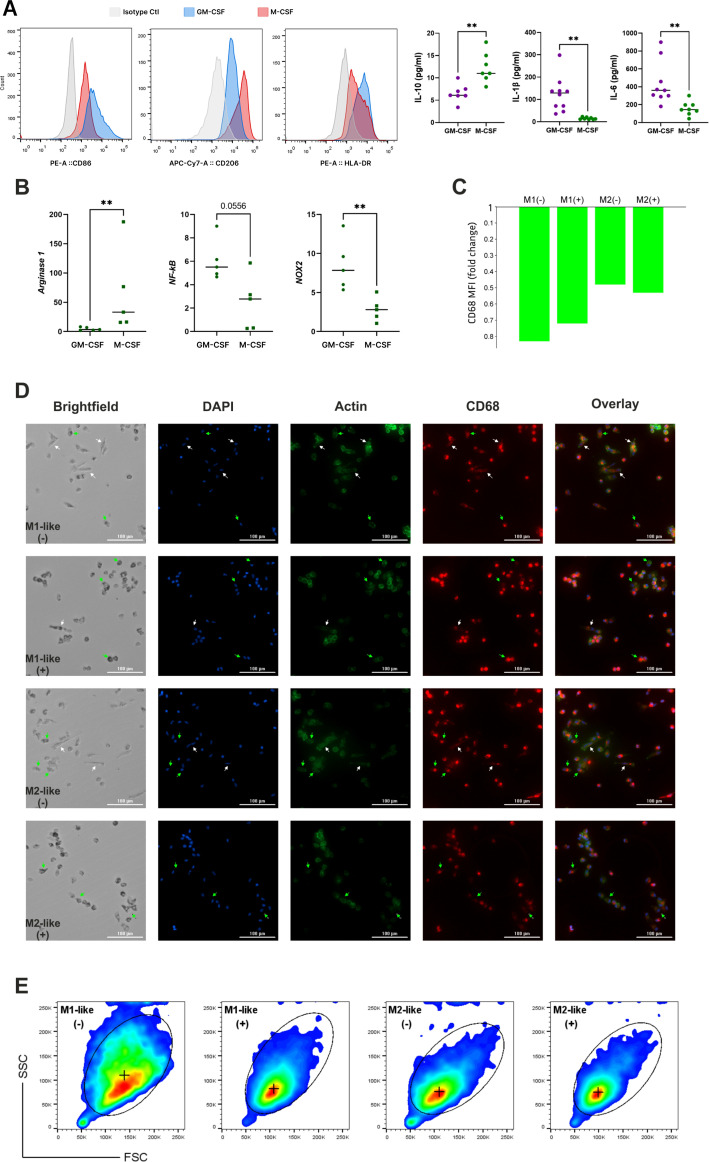
Establishing M1-like and M2-like macrophage subsets through GM-CSF and M-CSF-driven monocyte differentiation. **(A)** Validation of M1 and M2 polarization in cultured macrophages using flow cytometric evaluation of CD marker expression and cytokines production evaluated by ELISA; **(B)** mRNA expression levels of key factors typical for M1 and M2 macrophages; **(C)** CD68 expression measured with confocal microscopy and expressed as fold change of CD68 MFI level in differentiated (elongated) macrophages over non-differentiated (small spherical) cells, cultured under n-Glu (-) and h-Glu (+) conditions; **(D)** Immunofluorescent images of primary monocytes cultured 5 days with GM-CSF (M1-like macrophages) or M-CSF (M2-like macrophages) and stained for CD68 (red), F-actin stained with Phalloidin Alexa Fluor 488 (green) and counterstained with Hoechst (blue). Scale bar = 100 µm. **(E)** Flow cytometric (pseudo-color) plots showing the gating of M1-like and M2-likecells based on FSC/SSC. (+) median of FSC/SSC in gates. ** p<0.01.

The GM-CSF and M-CSF-derived macrophage model was employed to investigate the impact of short-term and long-term hyperglycemia. Primary monocytes were exposed to n-Glu and h-Glu conditions in the presence of GM-CSF or M-CSF for a duration of 7 days. Over the 5^th^ day cultivation period, monocytes treated with GM-CSF and M-CSF differentiated into M1-like and M2-like macrophages, respectively. Comparison of cell surface molecule expression and cytokine production by GM-CSF and M-CSF-treated monocytes confirmed a shift of GM-CSF-treated cells towards M1-like macrophages and M-CSF-treated cells towards M2-like macrophages. For the phenotypical characterization of differentiated macrophages, the expression of M1 (HLA-DR, CD86) and M2 (CD206, CD36) antigens were evaluated. CD86 (1.9-fold), CD206 (1.6-fold), and HLA-DR (1.8-fold) were differentially expressed between GM-CSF- and M-CSF-treated cells (**Figure 1A**). Cytokine levels in supernatants obtained from differentiated cells after 5 days of cultivation demonstrated that GM-CSF-treated cells produced significantly higher amounts of the IL-1β (8-fold) and IL-6 (2.8-fold), and lower concentrations of IL-10 (1.9-fold) (**Figure 1A**). Additionally, mRNA levels of genes typical for M1 (*NF-kB*, *NOS2*) and M2 (*Arginase-1*) macrophages were corresponding to the GM- and M-differentiated monocytes, correspondingly (**Figure 1B**). Particularly, analysis of basal mRNA expression signatures of M1- and M2-like macrophages revealed higher levels of *NF-kB* (*p=0.055*) and *NOX2* (*p<0.01*) in GM-CSF-treated cells compared with M-CSF-treated ones, while expression of *Arginase 1* was in opposite higher in M-CSF-treated cells (*p<0.01*) (**Figure 1B**). Thus, the scheme using GM-CSF and M-CSF as differential factors for the polarization of monocytes to M1-like and M2-like cells provided a reliable model to study macrophage responses under hyperglycemic conditions. This polarization model allowed us to examine the distinct effects of LPS stimulation and endotoxin tolerance on these two macrophage subsets.

In order to visualize the morphology of naïve GM-CSF or M-CSF-treated cells, we stained the cells for nuclei, actin, and CD68. Following cell differentiation with growth factors, the two resulting cell populations showed distinct morphological features (**Figures 1C, D**). Particularly, we observed that GM-CSF or M-CSF-differentiated macrophages displayed different cell morphologies, as has also been reported by other groups (17, 18). Both GM-CSF or M-CSF- cells appeared as heterogeneous cell populations with a mixture of 3 different morphologies: i) small cells with spherical shape; ii) small-middle size cells with high granularity; iii) large elongated (differentiated) cells. The difference between GM-CSF or M-CSF- cells is in the relative number/percentage of differentiated cells, as well as their lengths and area. Particularly, the percentage of differentiated cells in GM-CSF macrophages was lower (16%) than in M-CSF macrophages (24%). Conversely, GM-CSF macrophages have a higher number of highly granular cells compared to M-CSF macrophages (8.8% *vs.* 4.7%, correspondingly). In M-CSF-differentiated cells length and area were higher in comparison with GM-CSF macrophages (length 54 ± 24.2 μm *vs.* 39 ± 8.4 μm, area 427 ± 109.6 μm^2^ *vs.* 375.5 ± 136.3 μm^2^, respectively). The length and area sizes in small spherical GM-CSF and M-CSF-treated cells were not different. This data was confirmed by measuring forward scatter (FSC) and side scatter (SSC) using flow cytometry (**Figure 1E**).

Based on the phenotypic, molecular, and morphological characterization, GM-CSF and M-CSF-treated cells were validated as representative models of M1-like and M2-like macrophage subsets, respectively. Accordingly, we refer to these populations as M1-like and M2-like macrophages throughout the remainder of the manuscript.

### Long hyperglycemia results in distinct morphological changes in M1- and M2-like macrophages

As shown in **Figure 1D**, elongated/differentiated cells (white arrows) are present in both the M1- and M2-like macrophages under n-Glu conditions, while under h-Glu conditions, the number of differentiated M1-like cells was reduced by 2-fold and M2-like cells by 3.5-fold. Both M1-like and M2-like cells in h-Glu conditions exhibited a large number of heterogeneously shaped, highly granulated cells (green arrows, 17% *vs.* 18%). In contrast, the cells under n-Glu conditions displayed fewer numbers of cells with less granulation (8.8% *vs.* 4.7%). Additionally, a difference in size between cell types was observed: under both n-Glu and h-Glu conditions, differentiated M2-like cells are significantly larger in terms of area and length compared to M1-like cells. To verify macrophage identity in the differentiated cell cultures, we used CD68, a lysosomal-associated membrane glycoprotein from the LAMP family and a well-established pan-macrophage marker. To our knowledge, there is no clear evidence in the literature regarding differential CD68 expression between M1 and M2 macrophages. However, our results showed a slightly higher expression in GM-CSF-treated cells compared to M-CSF-treated cells. CD68 expression varied between cells depending on their differentiation status. Specifically, in differentiated cells, CD68 was distributed throughout the cytoplasm, with low expression levels observed (**Figure 1C**). In contrast, non-differentiated round-shaped cells exhibited higher levels of CD68, which was more compactly localized (**Figure 1D**).

### High glucose induces opposite changes in the functionality and phenotype of M1- and M2-like cells

**Figure 2:**
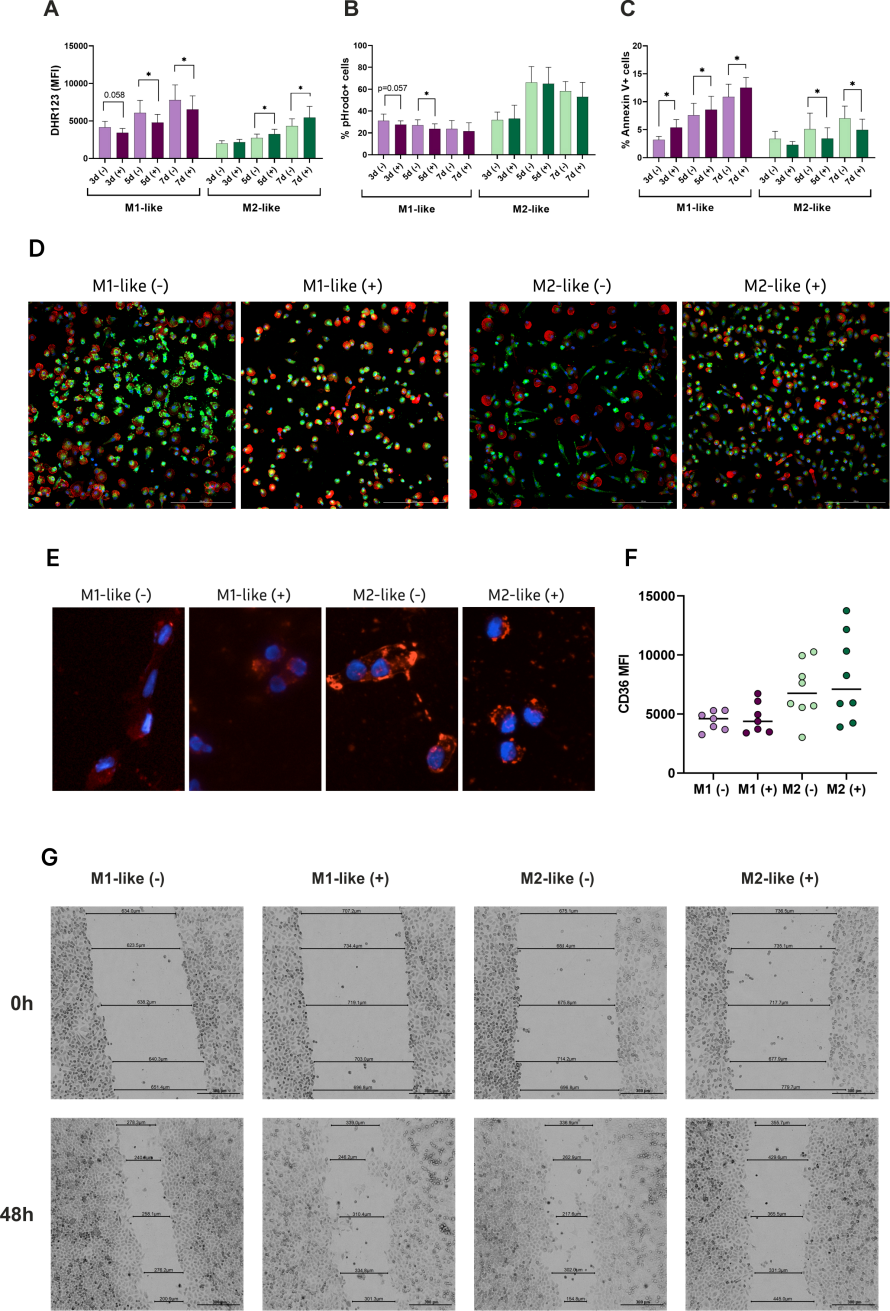
Hyperglycemia differentially drives functional properties of M1- and M2-like macrophages. Functional analyses were performed to assess the impact of glucose on: **(A)** ROS generation detected by DHR probe (n=9); **(B)** Phagocytic capacity of the cells cultured with pHrodo particles expressed as % of positive cells engulfed the particles (n=11); **(C)** Apoptotic rates assessed by Annexin V and propidium iodide (PI) staining (n=9); **(D)** Fluorescent images of NOS2 expression (green), actin (red), Hoechst (blue). **(E)** Fluorescent images of Oil Red O staining for oxLDL uptake by M1- and M2-like macrophages. The cells accumulated lipids in the cytoplasm after 24 hours of the treatment; **(F)** Basal expression levels of CD36 on M1- and M2-like macrophages before the treatment with oxLDL, measured with flow cytometry; **(G)** Wound healing of HeLa monolayer exposed by conditioned media form M1- and M2-like macrophages. Scratch distance and width closure was imaged at 0 h and 48h. The wound area at 0 h was qualified as 100%. *p<0.05.

Both short-term and long-term glycaemia had different effects on reactive oxygen species (ROS) production in M1- and M2-like macrophages. M1 cells showed a significant reduction in ROS activity when exposed to h-Glu throughout the entire cultivation period, whereas M2 cells exhibited increased ROS activity under h-Glu conditions (**Figure 2A**). Notably, under n-Glu conditions, M1 cells initially generated more ROS than M2 cells, which aligns with findings in the literature (19, 20).

Macrophages are renowned for their role as professional phagocytes and antigen presenting cells. We studied the phagocytic capacity of the cells using pHrodo particles. M2-like cells demonstrated a more pronounced phagocytic activity which was increasing over the cultivation period. Under h-Glu conditions, the phagocytic ability of M1-like macrophage was suppressed on days 3 and 5. M2-like cells retained their phagocytic function regardless of glucose conditions (**Figure 2B**).

Apoptotic rates in the studied cells also demonstrated opposing effects in the presence of high glucose. Specifically, there was an acceleration of apoptotic rates in M1-like cells, whereas M2-like cells displayed reduced apoptotic rates during the whole cultivation period (**Figure 2C**).

To assess whether high-glucose conditions influence NOS2 expression in a polarization-dependent manner, we examined the expression of inducible nitric oxide synthase (NOS2), a hallmark of classically activated (M1-like) macrophages, using fluorescence microscopy. NOS2 is typically upregulated in response to pro-inflammatory stimuli and plays a central role in nitric oxide (NO) production, contributing to the inflammatory capacity of M1 macrophages. As shown in **Figure 2D**, the highest NOS2 expression was observed in M1-like macrophages under n-Glu conditions. However, h-Glu exposure markedly reduced NOS2 expression in M1-like cells, while the number of NOS2-expressing cells increased within the M2-like population.

We also studied the ability of the cells to engulf OxLDL. Our results supporting the pro-atherogenic properties of macrophages indicated an enhanced uptake of oxLDL by M2-like macrophages both in n-Glu and h-Glu conditions (**Figure 2E**). M1-like cells exhibited lower uptake of the lipids. Enhanced uptake of M2-like cells was associated with the basal increased expression levels of surface CD36 (albeit not significant) (**Figure 2F**). CD36 is a well-established scavenger receptor involved in the uptake of fatty acids and oxidized OxLDL (21). Its up-regulation, along with increased lipid uptake, suggests a certain role for CD36 in the process of lipid uptake.

Subsequently, we investigated the abilities of macrophages to repair tissue in the presence of high glucose using scratch wound healing assay. Given that cytokines are the main factors influencing reparation processes, we evaluated the impact of conditioned media from M1-like and M2-like macrophages on wound healing. The microscopic examination revealed that HeLa cell migration was significantly inhibited in the wells with conditioned media from M2-like cells at h-Glu levels, resulting in prolonged wound closure (54.5%) (**Figure 2G**). The best reparative rates were observed in the wells with conditioned media from M2-like cells in n-Glu. Conditioned media from these cells recovered monolayer of HeLa cells faster (73.3%) than other studied groups. The mean percentage of tissue recovery in M1-conditioned media under both n-Glu and h-Glu conditions was 66.3% and 69.7%, respectively.

**Figure 3:**
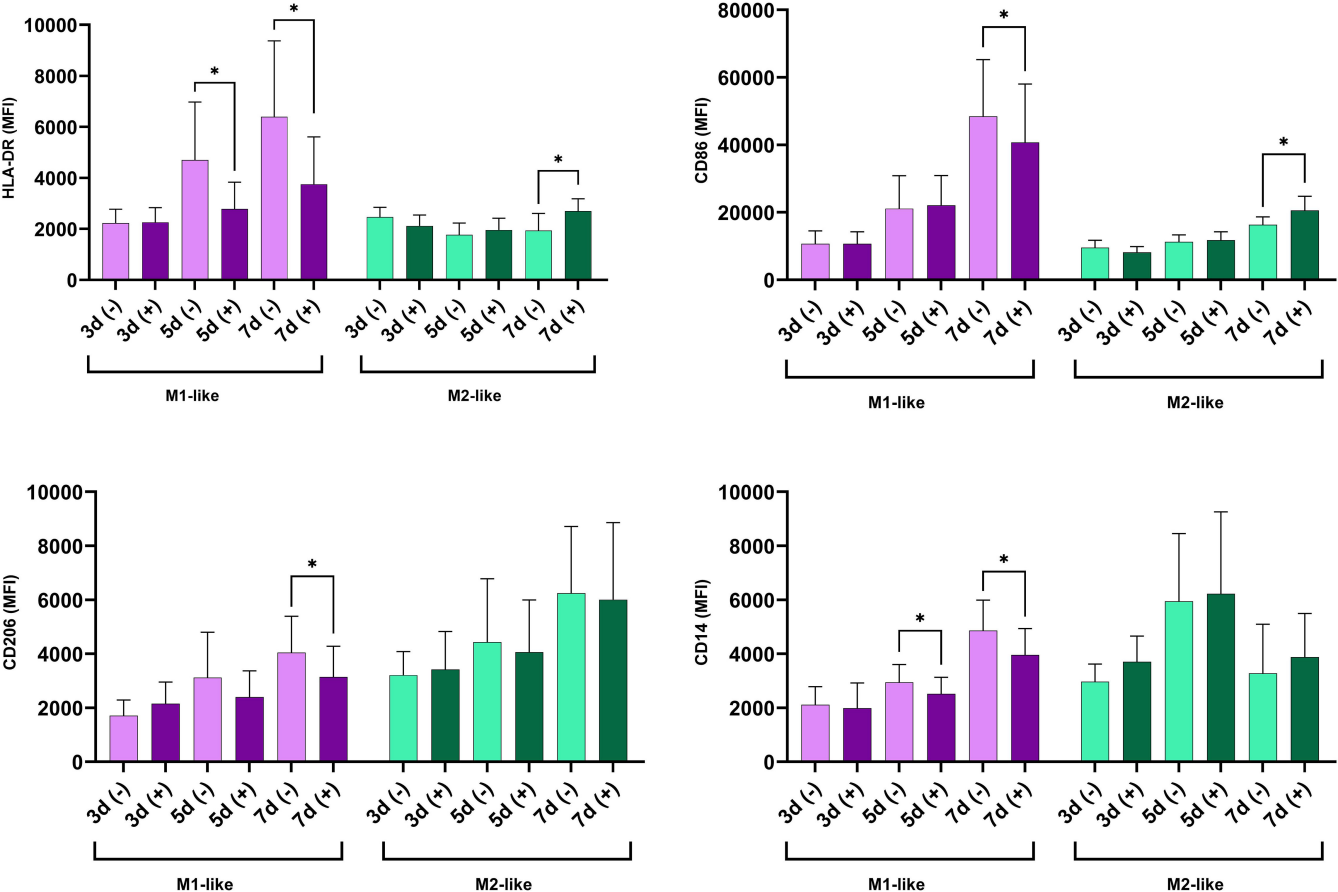
Time-dependent modulation of surface marker expression under hyperglycemic conditions in M1- and M2-like macrophages. The cells were cultured for indicated time in n-Glu (-) and h-Glu (+). Key surface markers were measured with flow cytometry (n=11). *p<0.05.

In line with the functional tests, immunophenotype of the cells revealed a different dynamic of cell activation between M1 and M2-like macrophages during both short-term and long-term glycemia. Upon long exposure to h-Glu, M1-like macrophages showed a diminished capacity for antigen presentation, as evidenced by reduced expression of HLA-DR on days 5 and 7. The decline in antigen presentation was accompanied by a reduction in co-stimulatory abilities, indicated by decreased CD86 expression on day 7. During extended exposure to h-Glu, M1-like macrophages also down-regulated expression of CD14 and CD206. M2-like macrophages reacted to prolonged hyperglycemia only on day 7 by increasing expression of HLA-DR and CD86 (**Figure 3**).

### Long-term exposure to high glucose suppressed the production of pro-inflammatory cytokines by M1 macrophages and increased their production by M2 macrophages

**Figure 4:**
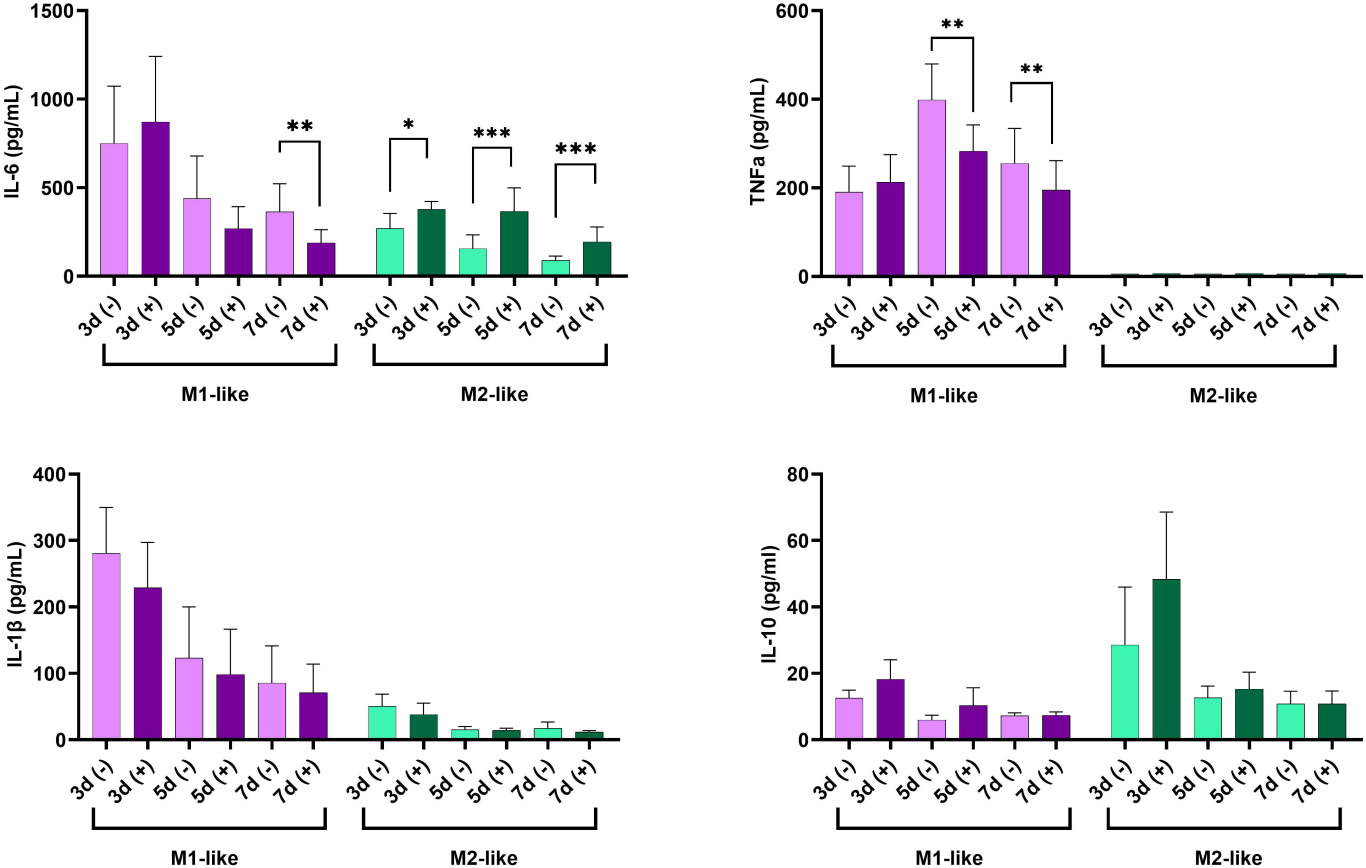
Time-dependent production of IL-6, TNF-α, IL-1β, and IL-10 in supernatants of cultured M1-like and M2-like macrophages under hyperglycemic conditions. *p<0.05; ** p<0.01; ***p<0.001.

Next, we investigated the dynamic of cytokine production (IL-1β, IL-6, TNF-α, IL-10) by M1- and M2-like macrophages cultured under n-Glu and h-Glu conditions on days 3, 5, and 7. It’s important to note that the media was replaced on the 3rd day of cell cultivation and then every 2^nd^ day thereafter. As a result, cytokines present in the supernatants collected on days 5 and 7 were representative of a 2-day cultivation period. As seen in **Figure 4**, M1-like cells cultured under n-Glu conditions displayed a classical cytokine profile characterized by higher levels of pro-inflammatory cytokines and lower levels of anti-inflammatory cytokines compared to M2-like macrophages. A notable observation was the trend of lower production of pro-inflammatory cytokines by M1-like cells during the cultivation process in h-Glu conditions compared to n-Glu. This trend was particularly evident on day 5 and 7, with significant reduction observed for TNF-α and on day 7 for IL-6. Although IL-1β levels showed a similar trend, statistical significance was not achieved. In contrast to M1-like cells, M2-like macrophages showed an increase in the production of IL-6 throughout all the studied days of cell cultivation. Additionally, M2-like cells produced low amounts of IL-1β and not detectable levels of TNF-α (**Figure 4**).

### Altered response to LPS by M1- and M2-like macrophages

Next, we investigated the response of the studied cells to LPS. Our results so far indicated that the M1-like and M2-like cells responded to long-term hyperglycemia differently. Macrophage polarization is regulated through the activation of transcription factors and signaling pathways downstream of receptors for various inducers (7). First, we aimed to analyze transcriptional activity of M1- and M2-related factors at basal level and after stimulation with LPS.

**Figure 5:**
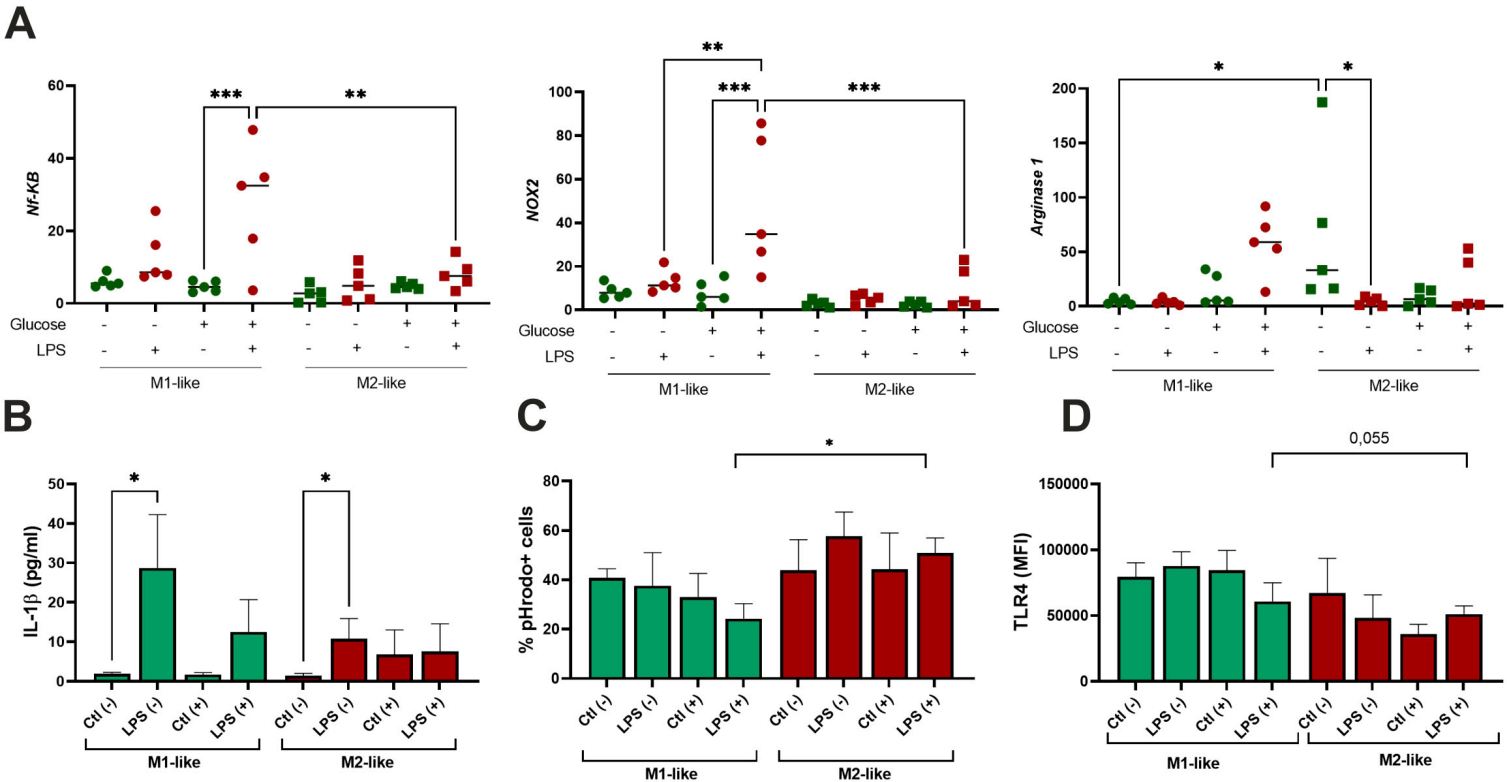
Effect of single-dose LPS stimulation on M1-like and M2-like macrophages. The cells were differentiated for 5 days and stimulated with 100 ng/ml LPS (n=5); **(A)** Quantitative RT-PCR analysis of *NF-kB, NOX2*, and *Arginase-1* mRNA levels in the cells treated with LPS for 4 hours. Expression values are normalized to *β-actin* and presented as relative fold changes; **(B)** Production of IL-1β by the cells exposed with LPS for 24 hours measured in culture supernatants; **(C)** Phagocytic activity of the cells treated with LPS for 24 hours and assessed using pHrodo™ Green Zymosan bioparticles; **(D)** Surface expression of TLR4 following LPS treatment and measured by flow cytometry. *p<0.05; ** p<0.01; ***p<0.001.

Despite being suppressed by hyperglycemia (described above), M1-cells retained pro-inflammatory activity as evidenced by the mRNA levels of *NF-kB* and *NOX2.* M2-like cells also up-regulated *NF-kB* and *NOX2*, but to a lesser extent, and these changes were not significant (**Figure 5A**). As expected, M2 cell marker *Arginase 1* was up-regulated by M2-like macrophages. Its expression was up-regulated by LPS under h-Glu conditions albeit not significant. While under n-Glu, in M2-like cells expression *Arginase 1* was down-regulated by LPS (**Figure 5A**).

IL-1β is a cytokine that potently promotes inflammation through activation of the transcription factor NF-κB. We aimed to analyze its production in the cells exposed to LPS. As appeared, the production of IL-1β was induced by LPS in both studied groups in n-Glu, while its increase in h-Glu was not significant (**Figure 5B**). As seen in **Figure 5C**, M2-like cells, in comparison to M1-like cells, exhibited slightly stronger phagocytic activity. However, a significant difference was observed only for LPS-treated cells under h-Glu conditions. M1-like cells showed slight decrease in the rates of phagocytosis in the presence of LPS at h-Glu conditions, while M2-like cells displayed an increase in the phagocytic activity (**Figure 5C**). TLR4 is a known sensor of Gram-negative bacterial infections through recognition of the LPS. Its surface expression was found to be down-regulated in M2-like cells at h-Glu conditions (**Figure 5D**), which was well associated with increased phagocytic abilities of the cells.

### High glucose affects endotoxin tolerance in M1-like macrophages

**Figure 6:**
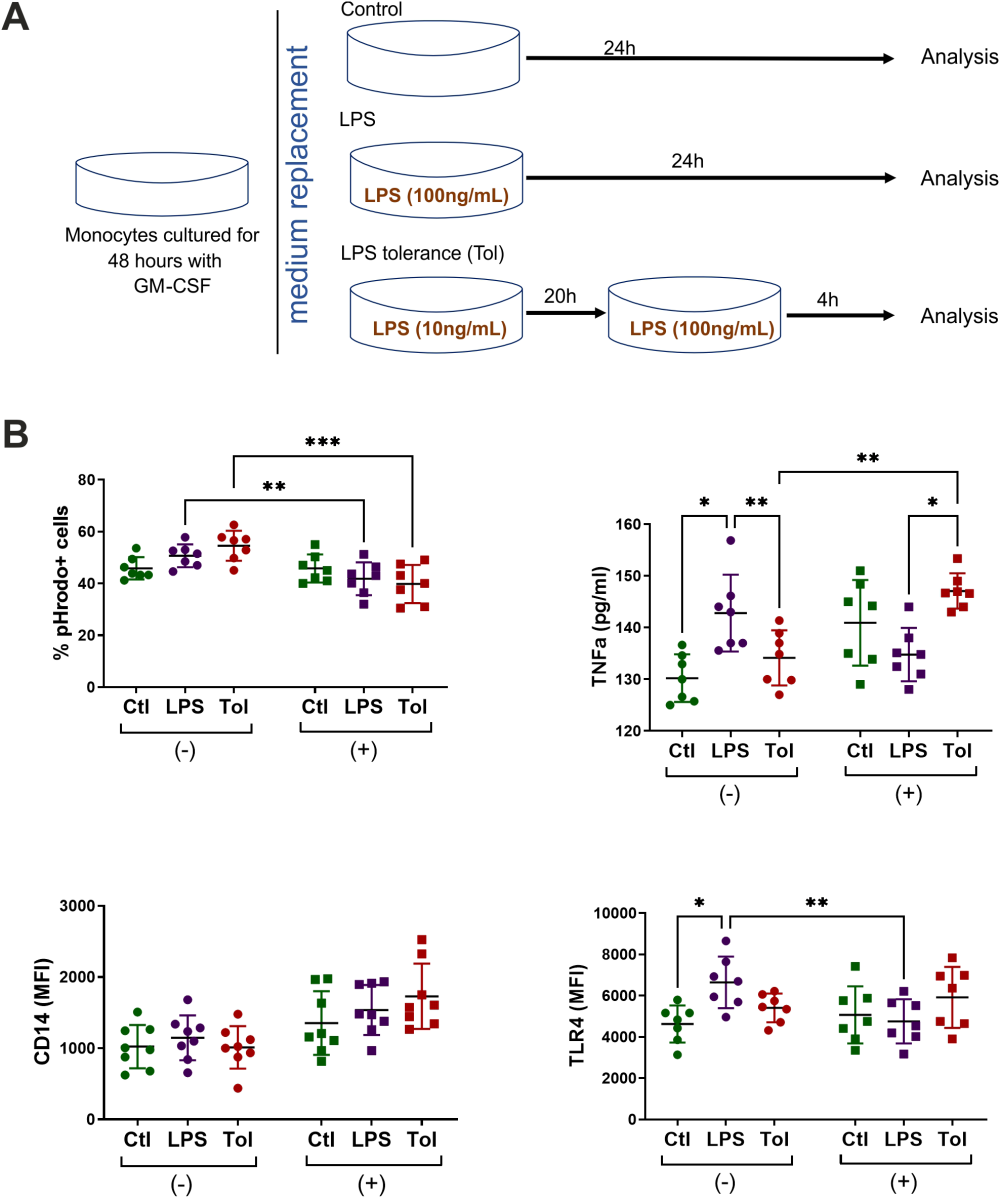
Tolerance model. **(A)** Schematic overview of LPS tolerance model using M1-like cells. Monocytes were cultured for 2 days with GM-CSF under n-Glu or h-Glu conditions and then exposed with the first dose of LPS (10ng/ml) for 20 hours. Following this, a repeated dose of LPS (100 ng/ml) was added for 4 hours (Tol). As controls, we used the cells cultured only with a single dose of LPS for 24 hours (LPS) and the cells cultured in media only (Ctl). Supernatants and the cells were collected to analyze surface expression of TLR4, phagocytosis and the production of TNFα; **(B)** Surface markers and phagocytic capacity of M1-like macrophages resulted from a single and repeated doses of LPS. Phagocytosis was assessed by adding pHrodo particles directly to the attached cells. Surface marker expression and phagocytosis were then analyzed on the detached cells. *p<0.05; ** p<0.01; ***p<0.001.

Next, we investigated whether macrophages responded differently to repeated exposures to LPS under n-Glu and h-Glu conditions. Repeated LPS stimulation was required to induce cell tolerance, a crucial regulatory mechanism in controlling inflammatory responses. “LPS tolerance” or “endotoxin tolerance” are well-known processes which are defined as a reduced capacity of the host to respond to a second or repeated LPS stimulation (22). Given that the suppression of LPS-induced cytokine production, particularly of TNF-α, is commonly acknowledged as a mechanism underlying the protective effect of endotoxin tolerance (23, 24), we exclusively used GM-CSF-treated cells to investigate endotoxin tolerance (**Figure 6A**). Our results revealed that TNF-α secretion from LPS-restimulated (tolerant) macrophages was significantly reduced compared to LPS-activated cells, suggesting successful establishment of the endotoxin tolerance model (25, 26). In h-Glu conditions, the cells showed re-programming of the processes involved in LPS tolerance, as evidenced by suppressed phagocytic activity and increased production of TNF-α (**Figure 6B**). It is worth mentioning that a single dose of LPS increased the production of TNF-α and expression of TLR4 under short-term n-Glu conditions, and didn’t induce significant changes under short-term h-Glu conditions.

## Discussion

The therapeutic potential of modulating metabolic reprogramming in immune cells is increasingly evident. Depending on the specific context, the ability to enhance or suppress M2 activation could offer therapeutic advantages. Exploring the mechanisms underlying macrophages reprogramming under high glucose conditions represents a crucial stride towards achieving this objective. Our study, for the first time, provides evidence that long-term hyperglycemia exerts divergent effects on the morphology, activation, and functionality of M1- and M2-like macrophages.

The results presented in this study indicate that hyperglycemia causes macrophage activation in a strikingly different fashion compared with the normoglycemic conditions. Long hyperglycemia reduced ROS activity in M1 macrophages, while M2 macrophages exhibited a gradual increase in ROS production over time, adopting characteristics reminiscent of M1 macrophages. In norm, M1 macrophages are characterized by glycolytic metabolism, the expression of inducible nitric oxide synthase (iNOS), and the production of pro-inflammatory cytokines. Elevated levels of glycolytic intermediates support nicotinamide adenine dinucleotide phosphate (NOX2) generation for nucleotide biosynthesis, ROS and lactate production (5, 7). M2 macrophages are characterized by increased mitochondrial respiration, production of anti-inflammatory cytokines, and high arginase-1 expression. The efficient microbicidal activity of macrophages relies on the sustained production of ROS and subsequent maturation of phagosomes. However, long hyperglycemia in our *in vitro* model reduced the effectiveness of bactericidal activity by M1 cells. In diabetes, the inflammatory response becomes prolonged over time where macrophages in the wound exhibit a failure to switch from a pro-inflammatory to a pro-healing phenotype suggesting that the pro-inflammatory state of macrophages may result in wide-ranging effects on the immune system. Recent studies showed that reduced ROS production improved wound healing in a model of diabetic mice (27, 28). Of note, M2 macrophages also express NOX2 oxidase, endowing them with the ability to generate ROS (29). Significantly increased ROS activity and reduced wound healing by M2 macrophages at h-Glu shown in our model further reinforcing abovementioned. Although initially beneficial, the wound healing process may turn detrimental when it persists long, resulting in the alteration of the extracellular matrix within the vessel wall. This is associated with an enhanced collagen deposition (30). ROS have been proposed as a mediator of the remodeling actions exhibited by M2 macrophages in fibrotic conditions, including lung fibrosis, muscular dystrophy, and the aortic stiffening linked to hypertension (31). Thus an increased pro-inflammatory activity of M2 macrophages caused by hyperglycemia might become a contributing factor for the ROS increase and reinforced pro-fibrotic functions of the cells.

ROS acts as an agonist of NF-κB signaling pathway initiating downstream inflammatory signaling pathways and leading to the release of inflammatory factors (32). Consistently, we observed up-regulated expression of NF-κB in M2-like cells in h-Glu which was associated with the increased expression of costimulatory CD86 and enhanced antigen-presentation by the cells. Chronic triggering of oxidative stress can cause aberrant responses to bacterial stimuli such as LPS. Despite being suppressed in hyperglycemia, M1 cells retain their ability to respond to a single dose LPS stimuli. Despite this, our results suggest that the reprogramming of M1 macrophages in response to metabolic changes involves complex regulatory mechanisms that affect their ability to respond to secondary stimuli. Secondary responses in innate immune cells can modify their capacity to react with either a stronger or weaker response compared to the primary one. As an example, repeated or persistent exposure of macrophages to a high dose of LPS ensures tolerance, preventing the expression of inflammatory genes (33). TLR4 signaling stimulated by LPS, drives macrophages to a preferentially M1 phenotype. Chronic signaling through the TLR4 pathway has been shown to induce various negative regulation which switch from an MyD88-dependent to a TRIF-dependent TLR4 pathway and switch macrophages to an immunosuppressive, endotoxin-tolerant phenotype. In our model, h-Glu appears to disrupt the establishment of endotoxin tolerance and potentially prime the induction of TNF-α in cells challenged with an additional dose of LPS. We previously showed that hypomethylation of the *IL1RN* and *NFKB1* gene promoters is associated with dysregulation of the IL-1β/IL-1Ra axis in type 2 diabetes (34). We hypothesized that hyperglycemia may act as an epigenetic modulator driving inflammatory priming of innate immune cells. These findings underscore the adaptation of the cells to ensure physiological adjustments to the increased levels of glucose, aiming to restore the cellular systems back to homeostatic states. However, side effects of these adjustments often occur that can lead to chronic inflammatory diseases (35, 36). Thus, the observed disruption of endotoxin tolerance and the priming of pro-inflammatory responses in hyperglycemic conditions highlight the adaptive but potentially harmful adjustments of macrophages to sustained high glucose levels.

Islet-resident macrophages may serve as initial detectors of systemic metabolic alterations and adapt their function and/or undergo local expansion in response. Tissues may contain mixed macrophage populations exhibiting a spectrum of functional and activation states. The transitions between the M1 and M2 phenotypes of macrophages are influenced by multiple factors, inflammation scenarios or disease progression (37). The inflammatory polarization of islet-associated macrophages is exacerbated during T2DM, shifting mainly towards M1-like phenotype (38, 39). The inflammatory polarization is further strengthened by the recruitment of circulating monocytes. This recruitment is observed to a much lesser extent compared to what is observed during T1DM. The results summarized so far indicate M1/M2 mixed polarization of T1DM macrophages (40, 41). Long-term hyperglycemia has the potential to essentially alter the paradigm of M1/M2 macrophage classification. Our study clearly shows that hyperglycemia suppresses proinflammatory program of M1 macrophages and increases pro-inflammatory potential of M2 macrophages. Macrophage populations often express mixed phenotypes in the course of various disease settings including neurodegenerative disorders, atherosclerotic plaques, and some tumors (10, 42). We suggest that characterization of tissue macrophages from the patients with metabolic deviations might represent a challenging task, complicating interpretation of their function. As an example, the novel M4, Mhem and M(Hb) macrophage subtypes described in atherosclerotic plaque formation and stability, which are polarized by CXCL4, heme and hemoglobin, respectively (43).

The mechanisms for macrophage reprogramming under hyperglycemia are currently largely unknown and may include multiple signaling molecules and pathways. Recent studies point to a key TRIM29-PERK axis in regulating macrophage function. TRIM29 suppresses macrophage inflammation by promoting NEMO degradation, inhibiting NF-κB and type I interferon signaling (44), and stabilizes PERK via SUMOylation to enhance ER stress and immunosuppressive programs (45). PERK itself supports M2 polarization by reprogramming mitochondrial metabolism (46). Thus, TRIM29 may coordinate both anti-inflammatory signaling and metabolic programming. Further investigation into this axis may reveal how metabolic stress promotes inflammation and tissue remodeling in metabolic disorders.

Re-polarization of macrophages is known phenomena, it was shown that M2 macrophages can be re-polarized into M1 macrophages following exposure to TLR ligands or IFNγ (47), whereas M1 macrophages can be reprogrammed to the macrophages with M2 phenotype with the agents that increase IL-10 level (48). Targeting colony-stimulating factor 1 (CSF1) and CSF1 receptor (CSF1R) for M2 macrophage reprogramming has been widely implemented in clinical trials for cancer therapy (49, 50). To ensure a comprehensive understanding of macrophage reprogramming under hyperglycemic conditions, future research should focus on elucidating the specific signaling pathways and molecular mechanisms involved. This could reveal potential therapeutic targets for modulating macrophage function in metabolic disorders and conditions characterized by chronic inflammation.

## Conclusion

In conclusion, long-term hyperglycemia exerts multifaceted effects on the functionality and polarization of macrophages, challenging the accepted paradigm of macrophage polarization in the context of metabolic disorders. Therapies aimed at modulating the inflammatory state of macrophages are increasingly recognized as valuable strategies to mitigate their detrimental effects. Our findings underscore the importance of carefully targeting macrophages in the management of metabolic disorders.

## Data Availability

The raw data supporting the conclusions of this article will be made available by the authors, without undue reservation.

## References

[1] Giri B Dey S Das T Sarkar M Banerjee J Dash SK. Chronic hyperglycemia mediated physiological alteration and metabolic distortion leads to organ dysfunction, infection, cancer progression and other pathophysiological consequences: An update on glucose toxicity. BioMed Pharmacother. (2018) 107:306–28. doi: 10.1016/j.biopha.2018.07.157, PMID: 30098549

[2] Ying W Fu W Lee YS Olefsky JM. The role of macrophages in obesity-associated islet inflammation and β-cell abnormalities. Nat Rev Endocrinol. (2020) 16:81–90. doi: 10.1038/s41574-019-0286-3, PMID: 31836875 PMC8315273

[3] Rohm TV Meier DT Olefsky JM Donath MY. Inflammation in obesity, diabetes, and related disorders. Immunity. (2022) 55:31–55. doi: 10.1016/j.immuni.2021.12.013, PMID: 35021057 PMC8773457

[4] Porta C Riboldi E Ippolito A Sica A. Molecular and epigenetic basis of macrophage polarized activation. Semin Immunol. (2015) 27:237–48. doi: 10.1016/j.smim.2015.10.003, PMID: 26561250

[5] Wculek SK Dunphy G Heras-Murillo I Mastrangelo A Sancho D. Metabolism of tissue macrophages in homeostasis and pathology. Cell Mol Immunol. (2022) 19:384–408. doi: 10.1038/s41423-021-00791-9, PMID: 34876704 PMC8891297

[6] Strizova Z Benesova I Bartolini R Novysedlak R Cecrdlova E Foley LK. M1/M2 macrophages and their overlaps - myth or reality? Clin Sci (Lond). (2023) 137:1067–93. doi: 10.1042/CS20220531, PMID: 37530555 PMC10407193

[7] Chen S Saeed AFUH Liu Q Jiang Q Xu H Xiao GG. Macrophages in immunoregulation and therapeutics. Signal Transduct Target Ther. (2023) 8:207. doi: 10.1038/s41392-023-01452-1, PMID: 37211559 PMC10200802

[8] Thiem K Keating ST Netea MG Riksen NP Tack CJ van Diepen J. Hyperglycemic memory of innate immune cells promotes *in vitro* proinflammatory responses of human monocytes and murine macrophages. J Immunol. (2021) 206:807–13. doi: 10.4049/jimmunol.1901348, PMID: 33431659

[9] Sun C Sun L Ma H Peng J Zhen Y Duan K. The phenotype and functional alterations of macrophages in mice with hyperglycemia for long term. J Cell Physiol. (2012) 227:1670–9. doi: 10.1002/jcp.22891, PMID: 21678423

[10] Moganti K Li F Schmuttermaier C Riemann S Klüter H Gratchev A. Hyperglycemia induces mixed M1/M2 cytokine profile in primary human monocyte-derived macrophages. Immunobiology. (2017) 222:952–9. doi: 10.1016/j.imbio.2016.07.006, PMID: 27492721

[11] Pavlou S Lindsay J Ingram R Xu H Chen M. Sustained high glucose exposure sensitizes macrophage responses to cytokine stimuli but reduces their phagocytic activity. BMC Immunol. (2018) 19:24. doi: 10.1186/s12865-018-0261-0, PMID: 29996768 PMC6042333

[12] Sousa ESA Queiroz LAD Guimarães JPT Pantoja KC Barros RS Epiphanio S. The influence of high glucose conditions on macrophages and its effect on the autophagy pathway. Front Immunol. (2023) 14:1130662. doi: 10.3389/fimmu.2023.1130662, PMID: 37122742 PMC10130370

[13] Xiu F Diao L Qi P Catapano M Jeschke MG. Palmitate differentially regulates the polarization of differentiating and differentiated macrophages. Immunology. (2016) 147:82–96. doi: 10.1111/imm.12543, PMID: 26453839 PMC4693883

[14] Verreck FA de Boer T Langenberg DM Hoeve MA Kramer M Vaisberg E. Human IL-23-producing type 1 macrophages promote but IL-10-producing type 2 macrophages subvert immunity to (myco)bacteria. Proc Natl Acad Sci U S A. (2004) 101:4560–5. doi: 10.1073/pnas.0400983101, PMID: 15070757 PMC384786

[15] Joshi S Singh AR Zulcic M Bao L Messer K Ideker T. Rac2 controls tumor growth, metastasis and M1-M2 macrophage differentiation *in vivo* . PloS One. (2014) 9:e95893. doi: 10.1371/journal.pone.0095893, PMID: 24770346 PMC4000195

[16] Lukic A Larssen P Fauland A Samuelsson B Wheelock CE Gabrielsson S. GM-CSF- and M-CSF-primed macrophages present similar resolving but distinct inflammatory lipid mediator signatures. FASEB J. (2017) 31:4370–81. doi: 10.1096/fj.201700319R, PMID: 28637652

[17] Heinrich F Lehmbecker A Raddatz BB Kegler K Tipold A Stein VM. Morphologic, phenotypic, and transcriptomic characterization of classically and alternatively activated canine blood-derived macrophages *in vitro* . PloS One. (2017) 12:e0183572. doi: 10.1371/journal.pone.0183572, PMID: 28817687 PMC5560737

[18] Rostam HM Reynolds PM Alexander MR Gadegaard N Ghaemmaghami AM. Image based Machine Learning for identification of macrophage subsets. Sci Rep. (2017) 7:3521. doi: 10.1038/s41598-017-03780-z, PMID: 28615717 PMC5471192

[19] Xu Q Choksi S Qu J Jang J Choe M Banfi B. NADPH oxidases are essential for macrophage differentiation. J Biol Chem. (2016) 291:20030–41. doi: 10.1074/jbc.M116.731216, PMID: 27489105 PMC5025689

[20] Yang Y Wang Y Guo L Gao W Tang TL Yan M. Interaction between macrophages and ferroptosis. Cell Death Dis. (2022) 13:355. doi: 10.1038/s41419-022-04775-z, PMID: 35429990 PMC9013379

[21] Park YM. CD36, a scavenger receptor implicated in atherosclerosis. Exp Mol Med. (2014) 46:e99. doi: 10.1038/emm.2014.38, PMID: 24903227 PMC4081553

[22] Seeley JJ Ghosh S. Molecular mechanisms of innate memory and tolerance to LPS. J Leukoc Biol. (2017) 101:107–19. doi: 10.1189/jlb.3MR0316-118RR, PMID: 27780875

[23] Lehner MD Morath S Michelsen KS Schumann RR Hartung T. Induction of cross-tolerance by lipopolysaccharide and highly purified lipoteichoic acid via different Toll-like receptors independent of paracrine mediators. J Immunol. (2001) 166:5161–7. doi: 10.4049/jimmunol.166.8.5161, PMID: 11290799

[24] Nahid MA Satoh M Chan EK. MicroRNA in TLR signaling and endotoxin tolerance. Cell Mol Immunol. (2011) 8:388–403. doi: 10.1038/cmi.2011.26, PMID: 21822296 PMC3618661

[25] del Fresno C García-Rio F Gómez-Piña V Soares-Schanoski A Fernández-Ruíz I Jurado T. Potent phagocytic activity with impaired antigen presentation identifying lipopolysaccharide-tolerant human monocytes: demonstration in isolated monocytes from cystic fibrosis patients. J Immunol. (2009) 182:6494–507. doi: 10.4049/jimmunol.0803350, PMID: 19414804

[26] López-Collazo E del Fresno C. Pathophysiology of endotoxin tolerance: mechanisms and clinical consequences. Crit Care. (2013) 17:242. doi: 10.1186/cc13110, PMID: 24229432 PMC4059412

[27] Hakami NY Dusting GJ Chan EC Shah MH Peshavariya HM. Wound healing after alkali burn injury of the cornea involves nox4-type NADPH oxidase. Invest Ophthalmol Vis Sci. (2020) 61:20. doi: 10.1167/iovs.61.12.20, PMID: 33079994 PMC7585390

[28] Feng C Yu B Song C Wang J Zhang L Ji X. Static magnetic fields reduce oxidative stress to improve wound healing and alleviate diabetic complications. Cells. (2022) 11:443. doi: 10.3390/cells11030443, PMID: 35159252 PMC8834397

[29] Kraaij MD Koekkoek KM van der Kooij SW Gelderman KA van Kooten C. Subsets of human type 2 macrophages show differential capacity to produce reactive oxygen species. Cell Immunol. (2013) 284:1–8. doi: 10.1016/j.cellimm.2013.07.006, PMID: 23916683

[30] Moore JP Vinh A Tuck KL Sakkal S Krishnan SM Chan CT. M2 macrophage accumulation in the aortic wall during angiotensin II infusion in mice is associated with fibrosis, elastin loss, and elevated blood pressure. Am J Physiol Heart Circ Physiol. (2015) 309:H906–17. doi: 10.1152/ajpheart.00821.2014, PMID: 26071547

[31] Lewis CV Vinh A Diep H Samuel CS Drummond GR Kemp-Harper BK. Distinct redox signalling following macrophage activation influences profibrotic activity. J Immunol Res. (2019) 2019:1278301. doi: 10.1155/2019/1278301, PMID: 31815149 PMC6877990

[32] Wu M Yang Z Zhang C Shi Y Han W Song S. Inhibition of NLRP3 inflammasome ameliorates podocyte damage by suppressing lipid accumulation in diabetic nephropathy. Metabolism. (2021) 118:154748. doi: 10.1016/j.metabol.2021.154748, PMID: 33675822

[33] Minassian AM Satti I Poulton ID Meyer J Hill AV McShane H. A human challenge model for Mycobacterium tuberculosis using Mycobacterium bovis bacille Calmette-Guerin. J Infect Dis. (2012) 205:1035–42. doi: 10.1093/infdis/jis012, PMID: 22396610 PMC3295601

[34] Margaryan S Kriegova E Fillerova R Smotkova Kraiczova V Manukyan G. Hypomethylation of IL1RN and NFKB1 genes is linked to the dysbalance in IL1β/IL-1Ra axis in female patients with type 2 diabetes mellitus. PloS One. (2020) 15:e0233737. doi: 10.1371/journal.pone.0233737, PMID: 32470060 PMC7259508

[35] Medvedev AE Lentschat A Kuhns DB Blanco JC Salkowski C Zhang S. Distinct mutations in IRAK-4 confer hyporesponsiveness to lipopolysaccharide and interleukin-1 in a patient with recurrent bacterial infections. J Exp Med. (2003) 198:521–31. doi: 10.1084/jem.20030701, PMID: 12925671 PMC2194174

[36] Divangahi M Aaby P Khader SA Barreiro LB Bekkering S Chavakis T. Trained immunity, tolerance, priming and differentiation: distinct immunological processes. Nat Immunol. (2021) 22:2–6. doi: 10.1038/s41590-020-00845-6, PMID: 33293712 PMC8020292

[37] Porcheray F Viaud S Rimaniol AC Léone C Samah B Dereuddre-Bosquet N. Macrophage activation switching: an asset for the resolution of inflammation. Clin Exp Immunol. (2005) 142:481–9. doi: 10.1111/j.1365-2249.2005.02934.x, PMID: 16297160 PMC1809537

[38] Cucak H Grunnet LG Rosendahl A. Accumulation of M1-like macrophages in type 2 diabetic islets is followed by a systemic shift in macrophage polarization. J Leukoc Biol. (2014) 95:149–60. doi: 10.1189/jlb.0213075, PMID: 24009176

[39] Calderon B Carrero JA Ferris ST Sojka DK Moore L Epelman S. The pancreas anatomy conditions the origin and properties of resident macrophages. J Exp Med. (2015) 212:1497–512. doi: 10.1084/jem.20150496, PMID: 26347472 PMC4577842

[40] Eguchi K Nagai R. Islet inflammation in type 2 diabetes and physiology. J Clin Invest. (2017) 127:14–23. doi: 10.1172/JCI88877, PMID: 28045399 PMC5199688

[41] Wang YJ Traum D Schug J Gao L Liu C HPAP Consortium. Multiplexed *in situ* imaging mass cytometry analysis of the human endocrine pancreas and immune system in type 1 diabetes. Cell Metab. (2019) 29:769–783.e4. doi: 10.1016/j.cmet.2019.01.003, PMID: 30713110 PMC6436557

[42] Wang N Liang H Zen K. Molecular mechanisms that influence the macrophage m1-m2 polarization balance. Front Immunol. (2014) 5:614. doi: 10.3389/fimmu.2014.00614, PMID: 25506346 PMC4246889

[43] Nagenborg J Goossens P Biessen EAL Donners MMPC. Heterogeneity of atherosclerotic plaque macrophage origin, phenotype and functions: Implications for treatment. Eur J Pharmacol. (2017) 816:14–24. doi: 10.1016/j.ejphar.2017.10.005, PMID: 28989084

[44] Xing J Weng L Yuan B Wang Z Jia L Jin R. Identification of a role for TRIM29 in the control of innate immunity in the respiratory tract. Nat Immunol. (2016) 17:1373–80. doi: 10.1038/ni.3580, PMID: 27695001 PMC5558830

[45] Wang J Lu W Zhang J Du Y Fang M Zhang A. Loss of TRIM29 mitigates viral myocarditis by attenuating PERK-driven ER stress response in male mice. Nat Commun. (2024) 15:3481. doi: 10.1038/s41467-024-44745-x, PMID: 38664417 PMC11045800

[46] Raines LN Zhao H Wang Y Chen HY Gallart-Ayala H Hsueh PC. PERK is a critical metabolic hub for immunosuppressive function in macrophages. Nat Immunol. (2022) 23:431–45. doi: 10.1038/s41590-022-01145-x, PMID: 35228694 PMC9112288

[47] Ye J Xie C Wang C Huang J Yin Z Heng BC. Promoting musculoskeletal system soft tissue regeneration by biomaterial-mediated modulation of macrophage polarization. Bioact Mater. (2021) 6:4096–109. doi: 10.1016/j.bioactmat.2021.04.017, PMID: 33997496 PMC8091177

[48] Hyam SR Lee IA Gu W Kim KA Jeong JJ Jang SE. Arctigenin ameliorates inflammation *in vitro* and *in vivo* by inhibiting the PI3K/AKT pathway and polarizing M1 macrophages to M2-like macrophages. Eur J Pharmacol. (2013) 708:21–9. doi: 10.1016/j.ejphar.2013.01.014, PMID: 23375938

[49] Ao JY Zhu XD Chai ZT Cai H Zhang YY Zhang KZ. Colony-stimulating factor 1 receptor blockade inhibits tumor growth by altering the polarization of tumor-associated macrophages in hepatocellular carcinoma. Mol Cancer Ther. (2017) 16:1544–54. doi: 10.1158/1535-7163.MCT-16-0866, PMID: 28572167

[50] Bart VMT Pickering RJ Taylor PR Ipseiz N. Macrophage reprogramming for therapy. Immunology. (2021) 163:128–44. doi: 10.1111/imm.13300, PMID: 33368269 PMC8114216

